# Two new species of *Agaporomorphus* Guignot from Suriname (Coleoptera, Adephaga, Dytiscidae, Copelatinae)

**DOI:** 10.3897/zookeys.923.48337

**Published:** 2020-04-01

**Authors:** Kelly B. Miller

**Affiliations:** 1 Department of Biology and Museum of Southwestern Biology, University of New Mexico, Albuquerque, NM 87131-0001, USA University of New Mexico Albuquerque United States of America

**Keywords:** *
Agaporomorphus
*, diving beetle, South America, taxonomy, water beetle

## Abstract

Two new species are described in the Neotropical genus *Agaporomorphus* Guignot from Suriname: *A. hamatocoles***sp. nov.** and *A. tortus***sp. nov**. The species are included in a phylogenetic parsimony analysis of 13 morphological characters and all 12 known species. Two equally parsimonious arrangements are found with the only difference a rearrangement among the *A. knischi* clade. *Agaporomorphus tortus* belongs to the *A. dolichodactylus* group based on presence of an elongate, club-like lobe on the dorsal, basal surface of the male median lobe and long, subsinuate male mesotarsal claws and a small lobe at the apex of male mesotarsomere V. *Agaporomorphus hamatocoles* does not belong to a known species group and is phylogenetically isolated lacking synapomorphies characterizing the other groups, so the species is placed in its own species group. Male genitalia are illustrated for the new species and redrawn for all the species of the *A. dolichodactylus* group, and male mesotarsal claws are illustrated for *A. tortus* and redrawn for other members of the *A. dolichodactylus* group. New distribution records are reported for Suriname for the species *A. colberti* Miller and Wheeler and *A. pereirai* Guignot.

## Abstract (es)

Se describen dos nuevas especies en el género neotrópico *Agaporomorphus* Guignot de Surinam: *A. hamatocoles***sp. nov.** y *A. tortus***sp. nov**. Las especies se incluyen en un análisis de parsimonia filogenética de 13 carácteres morfológicos y las 12 especies conocidas. Se encuentran dos arreglos igualmente parsimoniosos, con la única diferencia de un reordenamiento entre el clado de *A. knischi*. *Agaporomorphus tortus* pertenece al grupo *A. dolichodactylus* basado en la presencia en el macho de un lóbulo alargado, que parece un palo en la superficie dorsal, al base del lóbulo mediano ; las largas garras mesotarsales subsinuosas; y un lóbulo pequeño en el ápice del mesotarsómero V. *Agaporomorphus hamatocoles* no pertenece a ningún grupo de especies conocidas, está aislada filogenéticamente y carece de sinapormorfias que caracterizan los otros grupos, así que la especie se coloca en su propio grupo . Los genitales de los machos se ilustran para las nuevas especies y también para todas las especies del grupo *A. dolichodactylus*. Las garras mesotarsales de los machosse ilustran para *A. tortus* y también para los otros miembros del grupo *A. dolichodactylus*. Se informan nuevos registros de distribución para Surinam para las especies *A. colberti* Miller y Wheeler y *A. pereirai* Guignot.

## Introduction

New species of *Agaporomorphus* Guingot have been discovered regularly as collecting has continued in new areas of South America (Miller 2001; 2005; Miller and Wheeler 2008; Miller 2014; Hendrich et al. 2015). Most recently, Hendrich et al.(2015) described a new species and its habitat as well the habitats of several other species in the genus in Peru. This was useful since the habitats for most species of this rare taxon are not known or well-known because specimens are often collected at lights at night. They appear to be generally associated with shaded forest pools in primary forest (Hendrich et al. 2015) or (*A. sharynae* Miller) in leaf-choked backwaters of shaded sandy streams (Miller 2014).

Species of *Agaporomorphus* are known only from lowland tropical South America (Miller 2001; 2005; Miller and Wheeler 2008; Libonatti et al. 2011; Torres et al. 2012; Miller 2014; Hendrich et al. 2015). The new species from Suriname described here bring the number in the genus to 12. *Agaporomorphus pereirai* Guignot was previously the only species known from Suriname. *Agaporomorphus* came out resolved as sister to *Madaglymbus* Shaverdo and Balke, a Malagasy genus, in the analysis by Shaverdo et al. (2008), but relationships among Copelatinae genera remain ambiguous. Members of *Agaporomorphus* have dramatic and unusually complex, asymmetrical male median lobes, and these two new species are no exception exhibiting some morphological structures unique among diving beetles. New records of other described species are also provided.

## Materials and methods

### Material

The new species are based on specimens from the Snow Entomological Collection, University of Kansas, Lawrence, Kansas, USA (**SEMC**, A.E.Z. Short, curator). The holotypes are deposited in the National Zoological Collection of Suriname, Paramaribo, Suriname (**NZCS**, P. Ouboter, curator). Paratypes are deposited in NZCS, SEMC, and the Museum of Southwestern Biology, Division of Arthropods, University of New Mexico, Albuquerque, New Mexico, USA (**MSBA**, K.B. Miller, curator). In addition, specimens of all other species in the genus, including the holotypes, were examined by the author except *A. julianeae* Hendrich, Apenborn, Burmeister, and Balke.

### Measurements

Measurements were acquired using an ocular scale on a Zeiss Discovery V8 dissecting microscope at 50× magnification. Measurements include:

**TL** total length;

**GW** greatest width across elytra;

**PW** greatest pronotal width;

**HW** greatest width of the head;

**EW** distance between the eyes;

**FL** greatest length of the metafemur;

**FW** greatest width of the metafemur.

The ratios TL/GW, HW/EW, and FL/FW were also calculated to provide an indication of overall shape, eye size, and leg segment size.

### Phylogeny

The new species were coded for the 12 characters described by Miller (2001; 2005; 2014) and Miller and Wheeler (2008). A new 13^th^ character was added (see below). Parsimony analysis was done using WinClada to organize character data (Nixon 2002) and Nona for analysis (Goloboff 1995). Phylogenetic methods are the same as in Miller (2001; 2005) and Miller and Wheeler (2008). The two new species described here are included in the analysis along with *A. julianeae* with its characters scored based on the published account (Hendrich et al. 2015). The character matrix is presented in Table 1.

**Figures 1–6.**
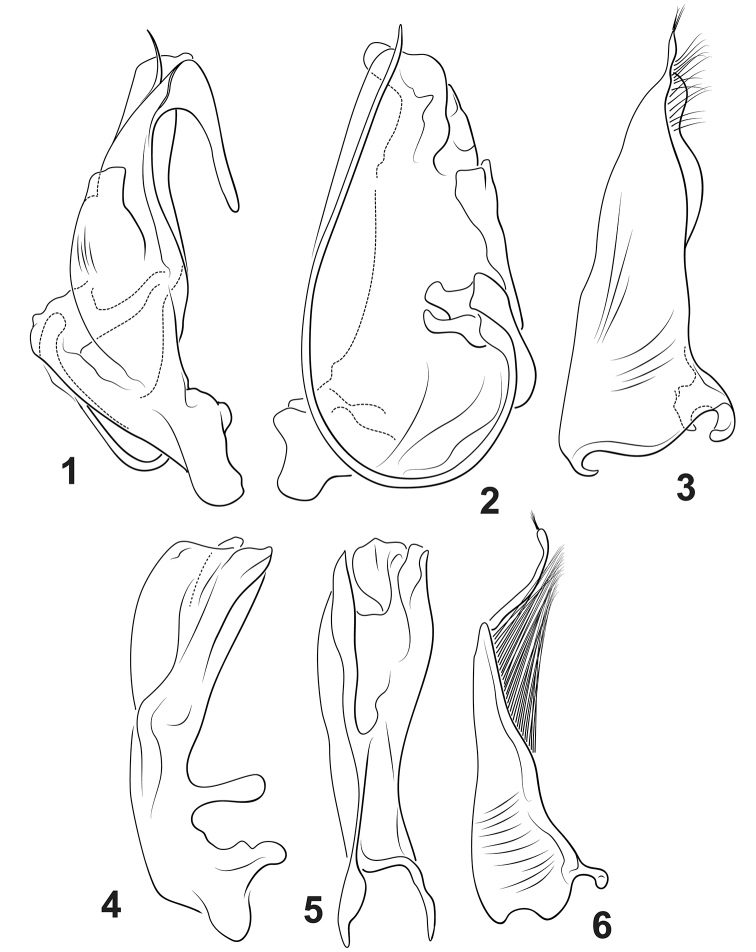
*Agaporomorphus* species, male genitalia. **1–3***A. hamatocoles***1** male median lobe, right lateral aspect **2** male median lobe, ventral aspect **3** male right lateral lobe, right lateral aspect **4–6***A. tortus***4** male median lobe, right lateral aspect **5** male median lobe, ventral aspect **6** male right lateral lobe, right lateral aspect.

Character 13. *Apical lobe on male lateral lobe*; (0) not extremely long and slender (Fig. 3), (1) extremely long and slender (Fig. 6). Specimens of *A. dolichodactylus*, *A. grandisinuatus*, *A. mecolobus* and *A. tortus* have the apical lobe on the male lateral lobe long and slender (e.g., Fig. 3). Other *Agaporomorphus*, including *A. hamatocoles*, have this lobe distinctly shorter (e.g., Fig. 6).

**Table 1.** Data matrix of assigned states of characters for 12 species of *Agaporomorphus* and generalized outgroup based on numerous examined taxa (e.g., *Copelatus* Erichson, *Madaglymbus* Shaverdo and Balke, *Lacconectus* Motschulsky, and *Exocelina* Broun species). Character 01 coded as additive (others binary). Characters match numbered characters from Miller (2001; 2005; 2014) and Miller and Wheeler (2008).

Species	0000000001111
	1234567890123
Outgroup	0000000000000
*A. hamatocoles*	0000000000000
*A. pereirai*	0000010000000
*A. knischi*	0000011111110
*A. tambopatensis*	0000001111010
*A. colberti*	0000001111110
*A. julianeae*	0000001101110
*A. silvaticus*	0000001000010
*A. sharynae*	0000011000010
*A. grandisinuatus*	1010000000001
*A. mecolobus*	2111100000001
*A. dolichodactylus*	2111100000001
*A. tortus*	2111100000001

## Taxonomy

### Agaporomorphus hamatocoles sp. nov.

Taxon classificationAnimaliaColeopteraDytiscidae

7B332F1C-B299-55FE-A4C3-8DFE59187768

http://zoobank.org/13D50A02-DB96-4BDF-B783-10546861859A

Figures 1–3 , 24 , 26

#### Type locality.

Suriname, Sipaliwini District, Sipaliwini Savannah Nature Reserve, Four Brothers Mountains, 2.005700N, 55.969151W, 337 m.

#### Diagnosis.

This species does not share many features with other members of the genus and does not have modified antennomeres, modified male mesotarsal claws or a lobe on the apex of mesotarsomere V, it lacks a stridulatory apparatus on the abdomen and metaleg, and lacks a triangular process at the apical margin of visible sternite V of the abdomen. Unique features of *A. hamatocoles* are the strongly hooked male median lobe (Fig. 1) and the elongate curved flagellum on the ventral surface of the male median lobe (Figs 1, 2). These features are diagnostic within *Agaporomorphus*.

#### Description.

***Measurements*** (*N* = 3). TL = 2.8–3.2 mm, GW = 1.4–1.6 mm, PW = 1.2–1.3 mm, HW = 0.8 mm, EW = 0.5–0.6 mm, FL = 0.7–0.8 mm, FW = 0.2–0.3 mm, TL/GW = 1.9–2.0, HW/EW = 1.5–1.6, FL/FW = 2.9–3.4. Body shape elongate oval, evenly and shallowly curved along lateral margins, curvature continuous between pronotum and elytron.

***Coloration.*** Head and pronotum dark orange. Elytron dark orange throughout except transverse basal band light orange. Ventral surface orange, similar in coloration throughout but legs distinctly lighter in color.

***Sculpture and structure.*** Head shiny, very finely microreticulate comprised of small isodiametric cells; eyes small (HW/EW = 1.5–1.6). Pronotum shiny, similar microreticulation to head; lateral margin slightly curved, extremely finely beaded, bead absent at anterior angle. Elytron with lateral margin shallowly curved; surface shiny, microreticulation extremely fine, apical half with numerous extremely fine punctures. Prosternum elongate, carinate, prosternal process short, strongly carinate medially. Metaventer and metaventral wings smooth and shiny, with very dense, extremely fine microreticulation. Metacoxa smooth and shiny, similar in microsculpture to metaventer; metacoxal lines distinct, region between metacoxal lines narrow medially; metafemur not unusually broadened (FL/FW = 2.9–3.4).

***Male genitalia.*** Median lobe exceptionally complex in shape, strongly asymmetrical; in lateral aspect broad basally, irregularly shaped, apically narrowed with apex dramatically hooked, curved anteriorly on dorsal surface with elongate apex directed posteriorly, curved portion elongate, slender and apically narrowly rounded (Fig. 1); in ventral aspect very broad, lateral margins broadly curved, with slender, long curved “flagellum” extending from left anteroventral region in broad curve along antero-ventral surface along left side to apex, apically sharply pointed (Fig. 2); lateral lobe in lateral aspect robust, apically narrowed, with slender apical lobe, with series of fine setae along apicodorsal margin (Fig. 3).

***Sexual dimorphism.*** Males have the pro-mesotarsomeres I-III distinctly broader than in females with enlarged ventral adhesive setae.

***Variation.*** The few specimens are quite similar to each other in coloration and other features.

#### Distribution.

This species is known only from southern Suriname (Fig. 24).

#### Habitat.

The type series was collected from “detrital pools.”

#### Discussion.

This species is quite unlike other species in the genus. The *A. knischi* group is characterized by somewhat similarly shaped male median lobes with a fringe of setae along the dorsal margin of each side and many of them have expanded male antennomeres and/or stridulatory devices on the abdomen and metalegs (Miller 2005; Miller and Wheeler 2008; Hendrich et al. 2015). The *A. dolichodactylus* group has an elongate process on the dorsal surface of the male median lobe and elongate, sinuate mesotarsal claws (Miller 2005). The *A. pereirai* group has none of these features, but the male median lobe has prominent angulate flanges on the ventral side apically and other autapomorphies (Miller 2005). The new species described here does not share any of these characteristic features and is phylogenetically isolated (Fig. 26, see below), so it is placed in its own group, the *A. hamatocoles* species group.

#### Etymology.

This species is named *hamatocoles*, from Latin *hamatus* for hooked and *coles* for penis for the unique shape of the hooked male median lobe in this species (Fig. 2).

#### Type material.

Holotype in NZCS, male labeled, “SURINAME: Sipaliwini District 2.005700N, 55.969151W, 337m Sipaliwini Savannah Nature Res. Four Brothers Mts, detrital pools, 31.iii.2017 leg. Short. SR17-0331-01D/ Holotype *Agaporomorphus hamatocoles* Miller, 2020 [red label with double black line border].” 3 paratypes labeled same as holotype except with “…/Paratype *Agaporomorphus hamatocoles* Miller, 2020 [blue label with black line border].”

### Agaporomorphus tortus sp. nov.

Taxon classificationAnimaliaColeopteraDytiscidae

C31B45B5-A1BB-5676-B57E-810842491BCC

http://zoobank.org/8FA75E35-E24C-47D5-9E82-939597462D54

Figures 4–6 , 16 , 17 , 24 , 26

#### Type locality.

Suriname, Sipaliwini District, Sipaliwini Savannah Nature Reserve, Four Brothers Mountains, 2°00.656'N, 55°59.070'W, 275 m.

#### Diagnosis.

**Figures 7–15.**
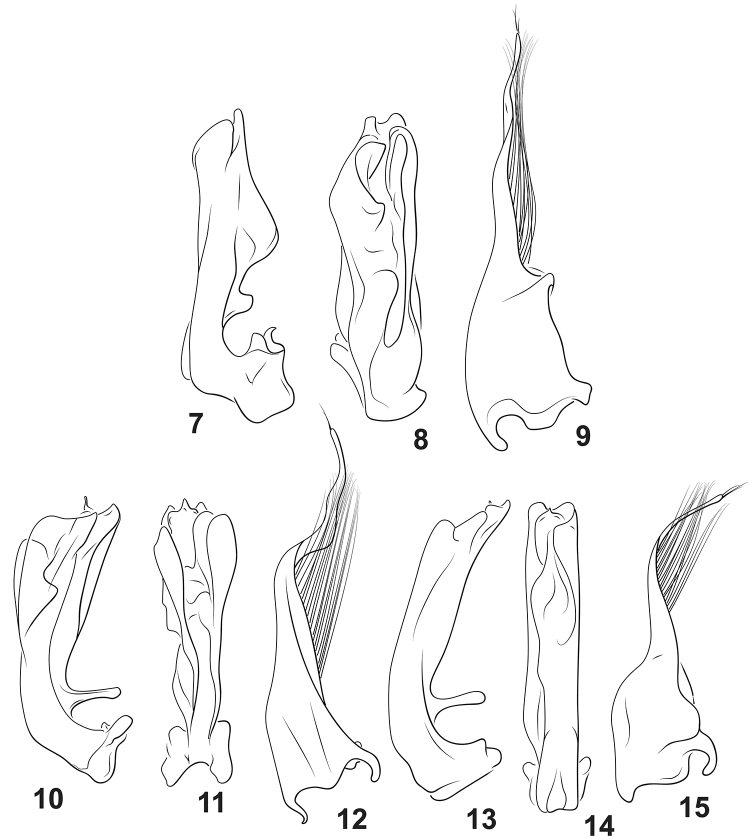
*Agaporomorphus* species, male genitalia. **7–9***A. grandisinuatus***7** male median lobe, right lateral aspect **8** male median lobe, ventral aspect **9** male right lateral lobe, right lateral aspect **10–12***A. mecolobus***10** male median lobe, right lateral aspect **11** male median lobe, ventral aspect **12** male right lateral lobe, right lateral aspect **13–15***A. dolichodactylus***13** male median lobe, right lateral aspect **14** male median lobe, ventral aspect **15** male right lateral lobe, right lateral aspect.

**Figures 16–23.**
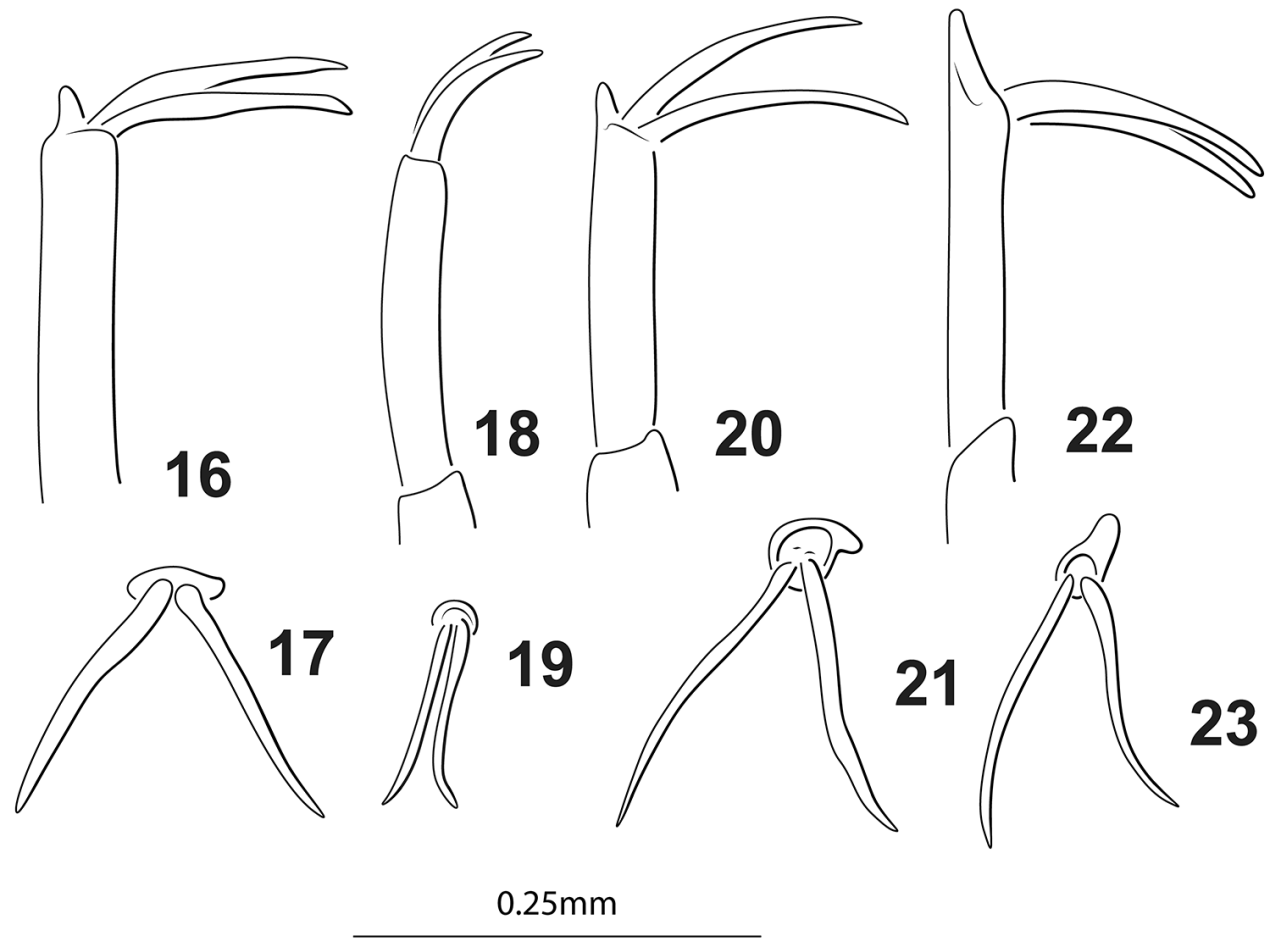
*Agaporomorphus* species, male mesotarsomere V and mesotarsal claws. **16, 17***A. tortus***16** anterior aspect **17** oblique lateral aspect **18, 19***A. grandisinuatus***18** anterior aspect **19** oblique lateral aspect **20, 21***A. mecolobus***20** anterior aspect **21** oblique lateral aspect **22, 23***A. dolichodactylus***22** anterior aspect **23** oblique lateral aspect.

This species is in the *A. dolichodactylus* species group which lacks characteristics of other species groups such as expanded male antennomeres, setae on the dorsal surface of the male median lobe, or stridulatory structures or triangular processes on the abdomen (Miller 2005). Like certain other members of the *A. dolichodactylus* species group, *A. tortus* has similar male genitalia (Figs 4–15) including an elongate process on the basal, dorsal surface of the male median lobe (Fig. 4), a lobe at the end of male mesotarsomere V (Figs 16, 17), and elongate and somewhat sinuate male mesotarsal claws (Figs 16, 17). From other species in the group this species differs in the shape of the male median lobe which is deeply asymmetrically emarginate apically in ventral aspect (Fig. 5) and with other distinctive shapes (Figs 4, 5).

#### Description.

***Measurements*** (*N* = 3). TL = 3.0–3.2 mm, GW = 1.5 mm, PW = 1.2–1.3 mm, HW = 0.8–0.9 mm, EW = 0.5 mm, FL = 0.7–0.8 mm, FW = 0.2–0.3 mm, TL/GW = 2.0–2.2, HW/EW = 1.6–1.7, FL/FW = 2.9–3.6. Body shape elongate oval, evenly and shallowly curved along lateral margins, curvature continuous between pronotum and elytron.

***Coloration***. Head, pronotum and elytron orange, similar in coloration throughout dorsal surface. Ventral surface orange, similar in coloration throughout but legs slightly lighter in color.

***Sculpture and structure.*** Head shiny, very finely microreticulate comprised of small isodiametric cells; eyes moderately large (HW/EW = 1.6–1.7). Pronotum shiny, similar microreticulation to head; lateral margin slightly curved, extremely finely beaded, bead absent at anterior angle. Elytron with lateral margin shallowly curved; surface shiny, microreticulation extremely fine, apical half with numerous extremely fine punctures. Prosternum elongate, carinate, prosternal process short, strongly carinate medially. Metaventer and metaventral wings smooth and shiny, with very dense, fine microreticulation. Metacoxa smooth and shiny, similar in microsculpture to metaventer; metacoxal lines distinct, region between metacoxal lines narrow medially; metafemur not unusually broadened (FL/FW = 2.9–3.6).

***Male genitalia.*** Median lobe complex in shape, asymmetrical; in lateral aspect narrow basally, broadened apically, apically broadly truncate with medial small lobe extending beyond truncation (Fig. 4); in ventral aspect broad apically, with complex arrangement of lobes and flanges, apicomedially with large, asymmetrical excavation between surfaces and distinctive deep apical emargination on left side of middle (Fig. 5); lateral lobe in lateral aspect robust basally, apically slender, with long, slender apical lobe, with long series of setae along dorsal margin (Fig. 6).

***Sexual dimorphism.*** Males protarsomeres I–III distinctly broader than in females with four large adhesive setae; females without expansion or adhesive setae. Male mesotarsomeres I–III broader than in females, not as strongly expanded as male protarsomeres I–III, male mesotarsomeres with four large ventral adhesives setae; apex of mesotarsomere V extended into small lobe on posterior margin of apex (Figs 16, 17), mesotarsal claws of male elongate, slightly sinuate (Figs 16, 17); females without these mesoleg modifications.

***Variation.*** There is some minor variation in intensity of coloration of the dorsal surface between specimens but this may be because some specimens are more teneral than others.

#### Distribution.

**Figures 24, 25.**
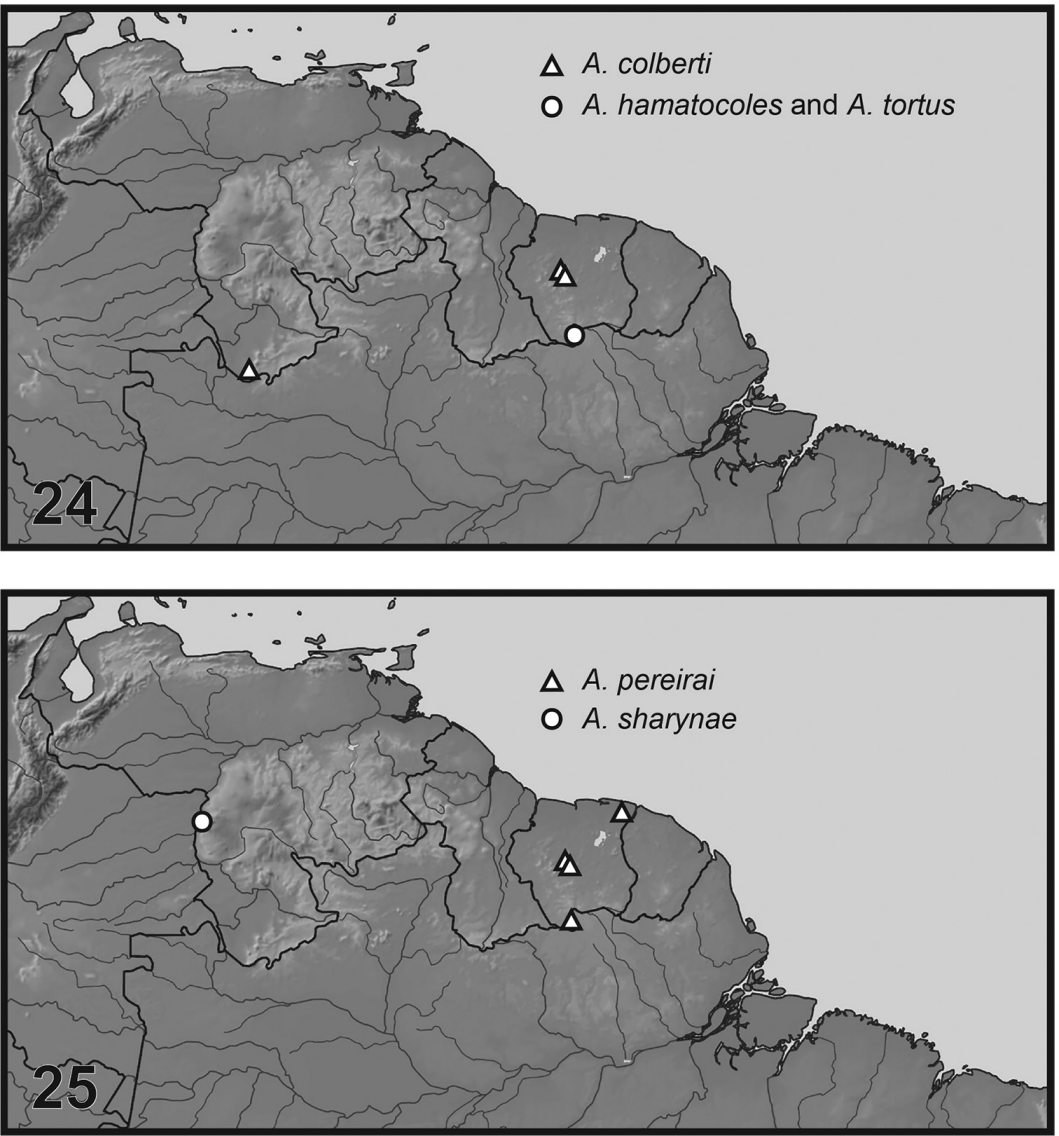
Known distributions of *Agaporomorphus* species of northern South America (*A. pereirai* also known from Brazil, not shown on map).

This species is only known from southern Suriname (Fig. 24).

#### Habitat.

The type series was collected from “vegetated pools in savanna.”

#### Discussion.

**Figure 26.**
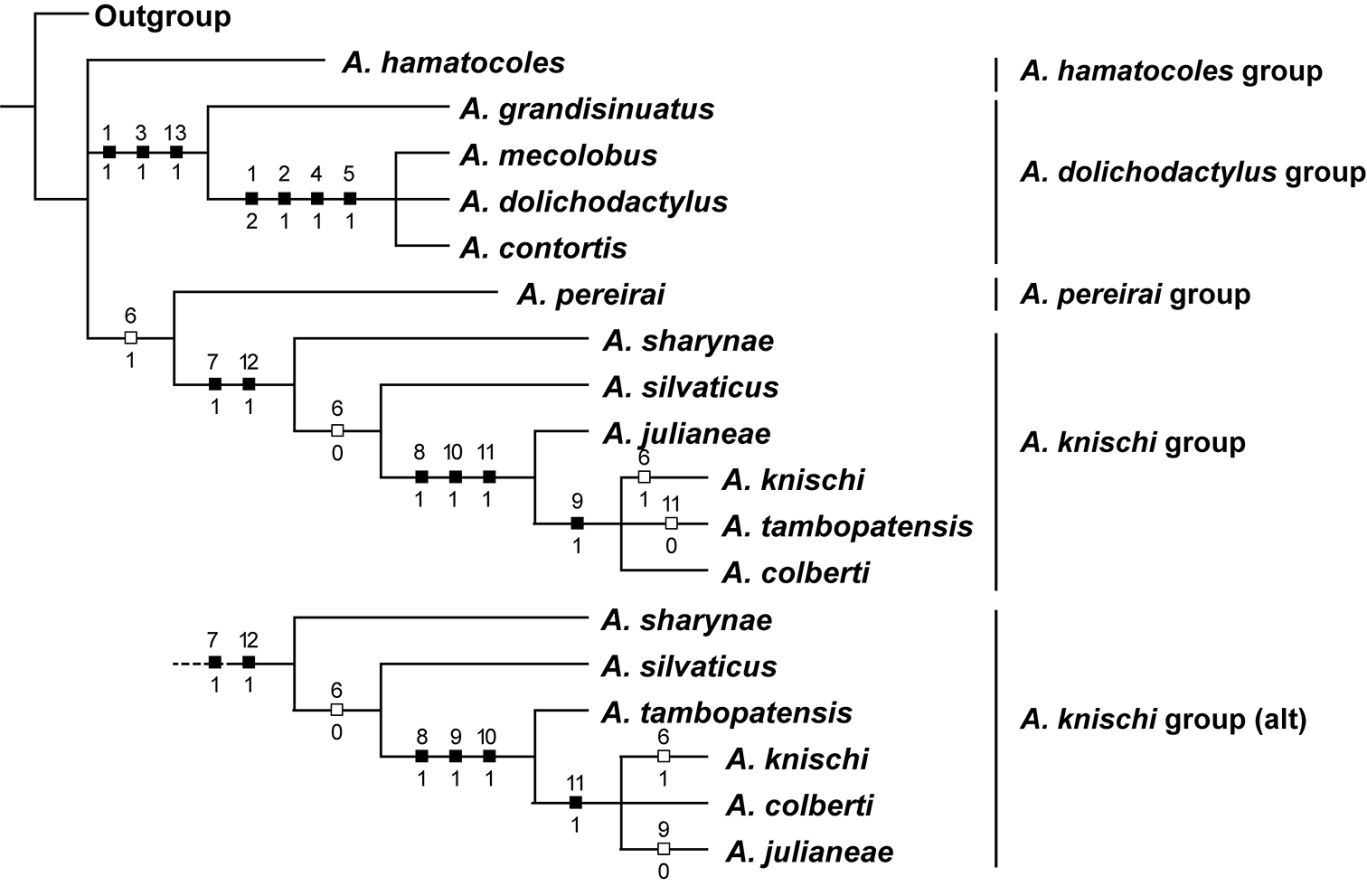
Two equally most parsimonious cladograms of *Agaporomorphus* species derived from parsimony analysis (L = 17, CI = 82, RI = 92): “alt” = alternative equally parsimonious configuration for *A. knischi* clade. Numbers above hatch marks refer to characters. Numbers below hatch marks refer to character state transformations. Characters mapped using “fast” or “acctran” optimization in WinClada (Nixon 2002).

This species belongs to the *A. dolichodactylus* group of *Agaporomorphus* of Miller (2005), and specifically close to *A. dolichodactylus* and *A. mecolobus* (Fig. 26, see below) based on the presence of a long lobe basally on the dorsal margin of the male median lobe (Fig. 4), a distinctive lobe on the apex of the male mesotarsomere V (Figs 16, 17), and male mesotarsal claws long and sinuate (Figs 16, 17). This is the first of the group known from northern South America (Fig. 24) with the other species in Brazil and Peru.

#### Etymology.

This species is named *tortus*, Latin for “twisted” for the complex shape of the male median lobe in this species (Figs 4, 5).

#### Type material.

Holotype in NZCS, male labeled, “Suriname: Sipaliwini District Sipaliwini Savanna Nature Res. 2°00.656'N, 55°59.070'W, 275 m vegetated pools in savanna 1.iv.2017; leg. A.E.Z. Short SR17-0401-01A/ SEMC1542796 KUNHM-ENT [barcode label]/ Holotype *Agaporomorphus tortus* Miller, 2020 [red label with double black line border].” 2 paratypes labeled same as holotype except […SEMC1542807…] and […SEMC1516119…] and paratype label, “…Paratype *Agaporomorphus tortus* Miller, 2020 [blue label with black line border].”

#### Phylogenetic results.

The parsimony analysis resulted in two equally parsimonious trees (L = 17, CI = 82, RI = 92) (Fig. 26). The trees comport well with previous results (Miller 2001; 2005; Miller and Wheeler 2008; Miller 2014) with three main clades characterized by specific distinctive synapomorphies. These correspond to the *A. dolichodactylus*-, *A. knischi*-, and *A. pereirai* groups of Miller (2001) with the exception of the new species *A. hamatocoles* (described above) which has an unresolved position in the tree because of absence of the synapomorphies shared among the other clades in the phylogeny (Fig. 26). The only difference between the trees is a rearrangement within the *A. knischi* clade (Fig. 26). The other new species, *A. tortus*, is resolved with the *A. dolichodactylus* clade based on presence of an elongate lobe on the dorsal base of the male median lobe (Figs 4, 19, 13, shorter and broadly rounded in *A. grandisinuatus*, Fig. 7). Specimens also have long, somewhat sinuate mesotarsal claws with a distinct lobe at the apex of mesotarsomere V (Figs 16, 17) (synapomorphy with *A. dolichodactylus* (Figs 20, 21) and *A. mecolobus* (Figs 22, 23) and a very long apical lobe on the male lateral lobe (Fig. 6), shared with other members of the *A. dolichodactylus* clade (Figs 9, 12, 15).

##### New records of other species of *Agaporomorphus*

***A. colberti* Miller and Wheeler** (Fig. 24). **Suriname**, Sipaliwini District, 3°47.479'N, 56°08.968'W, 320m, CSNR, nr Kappel airstrip, forest pools near Petromia Falls, 13 Aug 2013, Short, Bloom and Kadosoe, legs, SR13-0813-03A (3, KUNHM; SEMC1235490, SEMC1234094, SEMC1234095); Sipaliwini District, 3°47.479'N, 56°08.968'W, 320m, CSNR, nr Kappel airstrip, forested stream and stream pools, 24 Aug 2013, Short and Bloom, legs, SR13-0824-03A (2, KUNHM; SEMC1234951, SEMC0966126); Sipaliwini Dist, 3°55.600'N, 56°11.300'W, 600m, CSNR, Tafelberg Summit, nr Augustus Cr. Camp, pond on trail into Arrowhead basin, 16 Aug 2013, Short and Bloom, legs, SR13-0816-02A (A1, KUNHM: SEMC0930616).

These are the first records of *A. colberti* from Suriname with previous records from Venezuela (Miller and Wheeler 2008: fig. 24).

***A. pereirai* Guignot** (Fig. 25). **Suriname**, Sipaliwini Dist, 3°55.600'N, 56°11.300'W, 600m, CSNR, Tafelberg Summit, nr Augustus Cr. Camp, pond on trail into Arrowhead basin, 17 Aug 2013, Short and Bloom, legs, SR13-0817-01A (1, KUNHM: SEMC0965435, SEMC0965426, SEMC0965425, SEMC0965396, SEMC0965397); Sipaliwini Dist., 2°00.526'N, 55°58.572'W, 292m, Sipaliwini Savanna Nature Res, side pools of small clearwater stream in savannah, 20 Mar 2017, Short and Baca, legs, SR17-0330-02B (2, KUNHM; SEMC1541937, SEMC1541940); Sipaliwini Dist, 3°53.942'N, 56°10.849'W, 733m, SCNR, Tafelberg Summit, nr Caiman Cr camp, forest detrital pools, 19 Aug 2013, Short and Bloom, legs, SR13-0819-02A (1, KUNHM; SEMC0966170).

Previous records of this species are from Suriname (Cottica River, Moengo, Boven: fig. 25), and Matto-Grosso and Para, Brazil (Guignot 1957; Miller 2001).

##### Species list of the genus *Agaporomorphus*

***Agaporomorphus knischi* species group**

*A. colberti* Miller & Wheeler, 2008. Venezuela, Suriname

*A. julianeae* Hendrich, Apenborn, Burmeister, & Balke, 2013. Peru

*A. knischi* Zimmermann, 1921. Brazil, Peru, Bolivia

*A. sharynae* Miller, 2014. Venezuela

*A. silvaticus* Miller, 2005. Peru

*A. tambopatensis* Miller, 2005. Peru

***Agaporomorphus dolichodactylus* species group**

*A. dolichodactylus* Miller, 2001. Brazil, Bolivia

*A. grandisinuatus* Miller, 2001. Brazil, Peru

*A. mecolobus* Miller, 2001. Brazil

*A. tortus* sp. nov. Suriname

***Agaporomorphus hamatocoles* species group**

*A. hamatocoles* sp. nov. Suriname

***Agaporomorphus pereirai* species group**

*A. pereirai* Guignot, 1957. Brazil, Suriname

## Supplementary Material

XML Treatment for Agaporomorphus hamatocoles

XML Treatment for Agaporomorphus tortus
